# Establishment and validation of a ResNet-based radiomics model for predicting prognosis in cervical spinal cord injury patients

**DOI:** 10.1038/s41598-025-94358-7

**Published:** 2025-03-17

**Authors:** Zifeng Zhang, Ning Li, Yi Ding, Haowei Sun, Huilin Cheng

**Affiliations:** 1 School of Medicine, Southeast University, Nanjing, China; 2 Department of Neurosurgery, Zhongda Hospital, Southeast University, Nanjing, China; 3 Department of Neurosurgery, Nanjing medical university affiliated Suzhou Municipal Hospital, Suzhou, China

**Keywords:** Cervical spinal cord injury, Radiomics, Deep learning, Magnetic resonance imaging, Prognosis, Spinal cord diseases, Experimental models of disease

## Abstract

Cervical spinal cord injury (cSCI) poses a significant challenge due to the unpredictable nature of recovery, which ranges from mild paralysis to severe long-term disability. Accurate prognostic models are crucial for guiding treatment and rehabilitation but are often limited by their reliance on clinical observations alone. Recent advancements in radiomics and deep learning have shown promise in enhancing prognostic accuracy by leveraging detailed imaging data. However, integrating these imaging features with clinical data remains an underexplored area. This study aims to develop a combined model using imaging and clinical signatures to predict the prognosis of cSCI patients six months post-injury, helping clinical decisions and improving rehabilitation plans. We retrospectively analyzed 168 cSCI patients treated at Zhongda Hospital from January 1, 2018, to June 30, 2023. The retrospective cohort was divided into training (134 patients) and testing sets (34 patients) to construct the model. An additional prospective cohort of 43 cSCI patients treated from July 1, 2023, to November 30, 2023, was used as a validation set. Radiomics features were extracted using Pyradiomics and ResNet deep learning from MR images. Clinical factors such as age, smoking history, drinking history, hypertension, diabetes, cardiovascular disease, traumatic brain injury, injury site, and treatment type were analyzed. The LASSO algorithm selected features for model building. Multiple machine learning models, including SVM, LR, NaiveBayes, KNN, RF, ExtraTrees, XGBoost, LightGBM, GradientBoosting, AdaBoosting, and MLP, were used. Receiver operating characteristic (ROC) curves, calibration curves, and decision curve analysis (DCA) assessed the models’ performance. A nomogram was created to visualize the combined model. In Radiomics models, the SVM classifier achieved the highest area under the curve (AUC) of 1.000 in the training set and 0.915 in the testing set. Age, diabetes, and treatment were found clinical risk factors to develop a clinical model. The combined model, integrating radiomics and clinical features, showed strong performance with AUCs of 1.000 in the training set, 0.952 in the testing set and 0.815 in the validation set. And calibration curves and DCA confirmed the model’s accuracy and clinical usefulness. This study shows the potential of a combined radiomics and clinical model to predict the prognosis of cSCI patients.

**Supplementary Information:**

The online version contains supplementary material available at 10.1038/s41598-025-94358-7.

## Introduction

Patients with cervical spinal cord injury (cSCI) often vary with different degrees of paralysis^1–3^. Generally, patients with mild paralysis in the early stages tend to have better recovery potential^4,5^. However, even those with severe early paralysis can experience significant recovery, complicating prognostic predictions^6^. This uncertainty in prognosis prediction challenges for subsequent treatment decisions and rehabilitation choices^4–6^. To address this issue, the International Association of Neurorestoration introduced the Spinal Cord Injury Functional Rating Scale in 2019^7^. This comprehensive scale assesses the daily living activities and quality of life of cSCI patients, providing a quantitative measure for helping evaluate the prognosis of cSCI patients.

Over the past decade, deep learning has made remarkable advancements and become a core technology in the field of artificial intelligence^8–11^. This progress is mainly attributed to enhanced computational power, the availability of large datasets, and improved algorithms^10^. Deep learning has achieved groundbreaking success in image recognition, natural language processing, and speech recognition^8–11^. Among these advancements, the Residual Network (ResNet) technology stands out as a deep learning network architecture that addresses the issues of vanishing and exploding gradients in deep network training by introducing “skip connections.” These skip connections allow information to jump across layers, effectively bypassing one or more layers, which facilitates the training of deeper networks^12–14^. ResNet has demonstrated outstanding performance in various image classification tasks and become the foundation for many modern deep learning models^12,15–18^.

Currently, there is a relative scarcity of radiomics research focused on cSCI. In one retrospective study, 43 features were extracted from MRI images and clinical data to predict the 6-month post-injury ASIA grade in cSCI patients. Using the XGBoost algorithm, the model achieved an accuracy rate of 81.1%^19^. However, the study had notable limitations, as it relied on a limited set of predefined imaging features and did not include high-throughput radiomic features, potentially restricting its predictive performance. In contrast, another retrospective study by Okimatsu et al. employed deep learning to construct a Convolutional Neural Network (CNN) model for patients with cSCI within one month post-injury. The model was trained and validated on 294 sagittal T2-weighted MRI scans and utilized RF to predict neurological outcomes by incorporating patient age and initial ASIA grade. The model achieved accuracy, precision, recall, and F1 scores of 0.714, 0.590, 0.565, and 0.567, respectively^20^. While the results suggest the feasibility of using MRI images and machine learning for predicting neurological recovery in cSCI patients, this study had limitations as well. The images used for training included rectangular regions that encompassed not only the spinal cord but also surrounding structures, introducing significant noise and reducing the model’s overall performance. Moreover, the short follow-up period limited the assessment of long-term recovery.

We aimed to develop a composite model that integrates imaging and clinical features to predict the prognosis of cSCI patients six months post-injury and to evaluate the model’s application value. By combining radiomic features and deep learning techniques, we aspire to provide a more accurate and reliable prognostic tool for cSCI patients, thereby supporting clinical decision-making and optimizing rehabilitation strategies.

## Methods

### Study population

See Table 1 of patient baseline characteristics. This retrospective clinical cohort included 168 patients with cervical spinal cord injury (cSCI) who received treatment at Zhongda Hospital from January 1, 2018, to June 30, 2023, which was randomly divided into training and testing set. And the prospective clinical cohort included 43 patients with cSCI who received treatment at Zhongda Hospital from July 1, 2018, to November 30, 2023, as the validation set. Approval for the study was granted by the Zhongda Hospital Ethics Committee, with the ethical approval number 2023ZDSYLL137-P01.

**Table 1:** Patient baseline characteristics.

Category	Training(*n* = 134)		Testing(*n* = 34)	Validation(*n* = 43)		
IANR group	IANR ≥ 37(*n* = 107)	IANR<37(*n* = 27)	IANR ≥ 37(*n* = 27)	IANR<37(*n* = 7)	IANR ≥ 37(*n* = 31)	IANR<37(*n* = 12)
Male/Female(n)	89/18	22/5	9/18	3/4	7/24	3/9
Age($\:\stackrel{-}{x}$±s, years)	57.4 ± 11.0	65.0 ± 11.3	56.9 ± 11.4	70.4 ± 9.5	61.4 ± 12.0	56.7 ± 8.4
Smoking history(%)	32(30.0)	5(18.5)	4(14.8)	0(0)	11(35.5)	5(41.7)
Drinking history(%)	26(24.3)	4(14.8)	4(14.8)	0(0)	7(22.6)	5(41.7)
Hypertension(%)	31(30.0)	12(44.4)	10(37.0)	3(42.9)	17(54.8)	3(25.0)
Diabetes(%)	10(9.3)	7(25.9)	5(18.5)	2(28.6)	13(41.9)	10(83.3)
Cardiovascular disease(%)	11(10.3)	7(25.9)	2(7.4)	1(14.3)	7(22.3)	0(0)
Traumatic brain injury(%)	5(4.7)	4(14.8)	3(11.1)	1(14.3)	5(16.1)	2(16.7)
Injury site(%)						
C1-4	36(33.6)	11(40.7)	7(25.9)	2(28.6)	7(25.9)	2(28.6)
C5-T1	13(12.1)	7(25.9)	9(33.3)	2(28.6)	9(33.3)	2(28.6)
C1-T1	58(54.2)	9(33.3)	11(40.7)	3(42.9)	11(40.7)	3(42.9)
Treatment(%)						
Anterior approach	36(33.6)	4(14.8)	9(33.9)	3(42.9)	10(32.3)	5(41.7)
Posterior approach	42(39.3)	16(59.3)	13(48.1)	4(57.1)	21(67.7)	7(58.3)
Anterior&Posterior approach	2(1.9)	3(11.1)	1(3.7)	0(0)	0(0)	0(0)
Conservative treatment	27(25.2)	4(14.8)	4(14.8)	0(0)	0(0)	0(0)

The research was conducted in compliance with the Declaration of Helsinki. Informed consent was obtained from all participants, allowing the use of their data for research purposes. We defined a good prognosis group as having an IANR score ≥ 37, while a poor prognosis group was defined as having an IANR score < 37.

Inclusion criteria for the study were as follows: patients over 18 years of age, diagnosed with cSCI, who underwent standardized treatment, and proceeded with preoperative protocols. Exclusion criteria were patients with incomplete MRI sequences, spinal cord injury lesions that were not visible on MRI, those lost to follow-up, or those with incomplete clinical data. The dataset was randomly distributed in an 8:2 ratio, assigning it to the training set or the testing set.

### Radiomics model construction

MR images were obtained using two 3.0 T MRI scanners (Philips Ingenia 3.0T; Siemens MAGNETOM Verio 3.0 T) as shown in Supplementary Table 1. All patients were routinely scanned with sagittal T1WI, axial T1WI, coronal T1WI, sagittal T2WI, axial T2WI and coronal T2WI. Table 1 shows the parameters of the selected sequences of each MRI scanner.

We employed two methods to extract radiomic features. First, we delineated the injury lesion as the volume of interest (VOI) for handcrafted feature extraction. Second, we defined the region of interest (ROI) by cropping vertical rectangular areas on sagittal images at the injury level, encompassing the injury site and the anterior-posterior boundaries of the spinal canal, ensuring the region extended neither above nor below the lesion for deep learning feature extraction. Manual segmentations were carried out utilizing 3D Slicer software (https://www.slicer.org; version 5.0.3).

Radiologist A, with 7 years of experience in spine MRI diagnosis, performed delineations for all patients. To ensure robustness, radiologist B, who has 10 years of experience in spine MRI diagnosis, independently delineated a randomly selected subset of 50 patients. Both radiologists were blinded to the patients’ diagnoses. After a 2-month interval, radiologist A re-delineated the VOIs and ROIs for all patients. The intraclass correlation coefficient (ICC) was computed for each feature to evaluate inter-observer and intra-observer reliability, and features with an ICC below 0.75 were excluded.

For handcrafted feature extraction, features were extracted from the VOIs using an MRI feature analysis program in Pyradiomics (http://pyradiomics.readthedocs.io). For deep learning feature extraction, we utilized the ResNet152 algorithm, an advanced version of the ResNet algorithm introduced in 2015^21^. ResNet152, with its deeper architecture, enhances the model’s ability to capture intricate features from medical images, particularly in complex cases like spinal cord injury. We applied transfer learning to initialize the model with weights pre-trained on our datasets. This pre-training allowed the model to leverage general imaging features and adapt more efficiently to our specific cSCI dataset, leading to improved performance in capturing the relevant features of the injury. Transfer learning proved particularly beneficial in a medical imaging context, where labeled data can be limited, and specialized imaging patterns are crucial for accurate diagnosis and prognosis. Then we compressed the deep learning features through dimensionality reduction. Both handcrafted and deep learning features were standardized using the Z-score method. The least absolute shrinkage and selection operator (LASSO) was employed to filter out standardized features with non-zero coefficients, thus selecting and reducing the dimensionality of the fusion features to obtain the optimal subset.

Following LASSO feature screening, the final features were input into various machine learning models, including Logistic Regression (LR), Naive Bayes Classifier (NaiveBayes), Support Vector Machine (SVM), K Nearest Neighbors Classifier (KNN), Random Forest Classifier (RF), Extra Trees Classifier (ExtraTrees), eXtreme Gradient Boosting Classifier (XGBoost), Light Gradient Boosting Machine Classifier (LightGBM), Gradient Boosting Classifier (GradientBoosting), Adaptive Boosting Classifier (AdaBoosting), and Multi-Layer Perceptron Classifier (MLP) for predictive model construction. A 10-fold cross-validation was used to determine the final radiomics signature. Receiver operating characteristic (ROC) curves were plotted to assess the diagnostic performance of the predictive models, analyzing the area under the curve (AUC), diagnostic accuracy, sensitivity, and specificity.

### Clinical model construction

Age, smoking history, drinking history, hypertension, diabetes, cardiovascular disease, traumatic brain injury, injury site, and treatment (including anterior approach surgery, posterior approach surgery, anterior & posterior approach surgery and conservative treatment) were selected as clinical factors and analyzed for differences between groups. These clinical factors were fed into the LR model for clinical signature building.

### Combined model construction

To enhance the predictive performance, we constructed a combined model by integrating both radiomic features and clinical factors. We tested the combined model using the receiver operating characteristic (ROC) curves, as well as the AUC, sensitivity, specificity, and accuracy. By combining radiomics and clinical data, we aimed to improve the overall prognostic accuracy, providing a more comprehensive model for predicting neurological recovery in cSCI patients.

### Statistical analysis

Statistical analysis was conducted using SPSS (version 26.0), with significance set at *p* < 0.05. Continuous variables in the clinical data were assessed using independent t-tests or Mann-Whitney U tests, while categorical variables were analyzed using Fisher’s exact test or chi-square tests (Table 2).

**Table 2:** Demographic and clinical characteristics of study populations.

Characteristic	IANR ≥ 37(*n* = 134)	IANR<37(*n* = 34)	t/χ^2^	*P*
Male/Female(n)	107/27	26/8	0.039	0.844
Age($\:\stackrel{-}{x}$±s, years)	57.3 ± 11.1	66.1 ± 11.1	4.135	0.000
Smoking history(%)	36(26.9)	5(14.7)	1.564	0.211
Drinking history(%)	30(22.4)	4(11.8)	1.295	0.255
Hypertension(%)	41(30.6)	15(44.1)	1.664	0.197
Diabetes(%)	15(11.2)	9(26.5)	3.996	0.046
Cardiovascular disease(%)	13(9.7)	8(23.5)	3.561	0.059
Traumatic brain injury(%)	5(14.7)	8(6.0)	1.804	0.179
Injury site(%)			3.268	0.195
C1-4	43(32.1)	13(38.2)		
C5-T1	22(16.4)	9(26.5)		
C1-T1	69(51.5)	12(35.3)		
Treatment(%)			8.374	0.039
Anterior approach	45(33.6)	7(20.6)		
Posterior approach surgery	55(41.0)	20(58.8)		
Anterior&Posterior approach	3(2.2)	3(8.8)		
Conservative treatment	31(23.1)	4(11.8)		

## Results

**Fig. 1:**
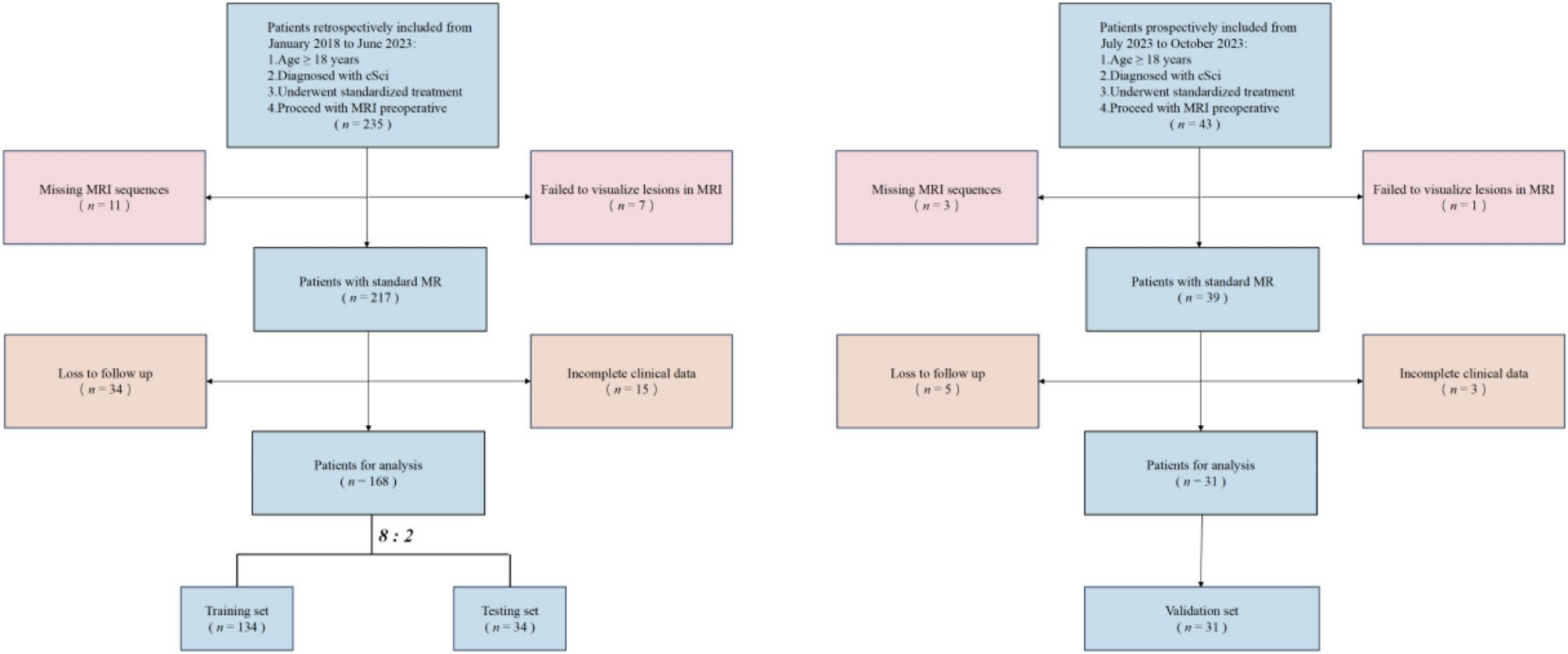
Flow chart demonstrating the inclusion and exclusion criteria for the study participants with cSCI.

A total of 168 patients were included in this study, randomly divided into a training set of 134 patients and an independent testing set of 34 patients at a ratio of 8:2. The patient flow through the study and the number of patients at each analysis stage are shown in Fig. 1. Patient characteristics for the cohort are detailed in Table 1. The definition of patient characteristics is shown in Supplementary Table 2.

**Fig. 2:**
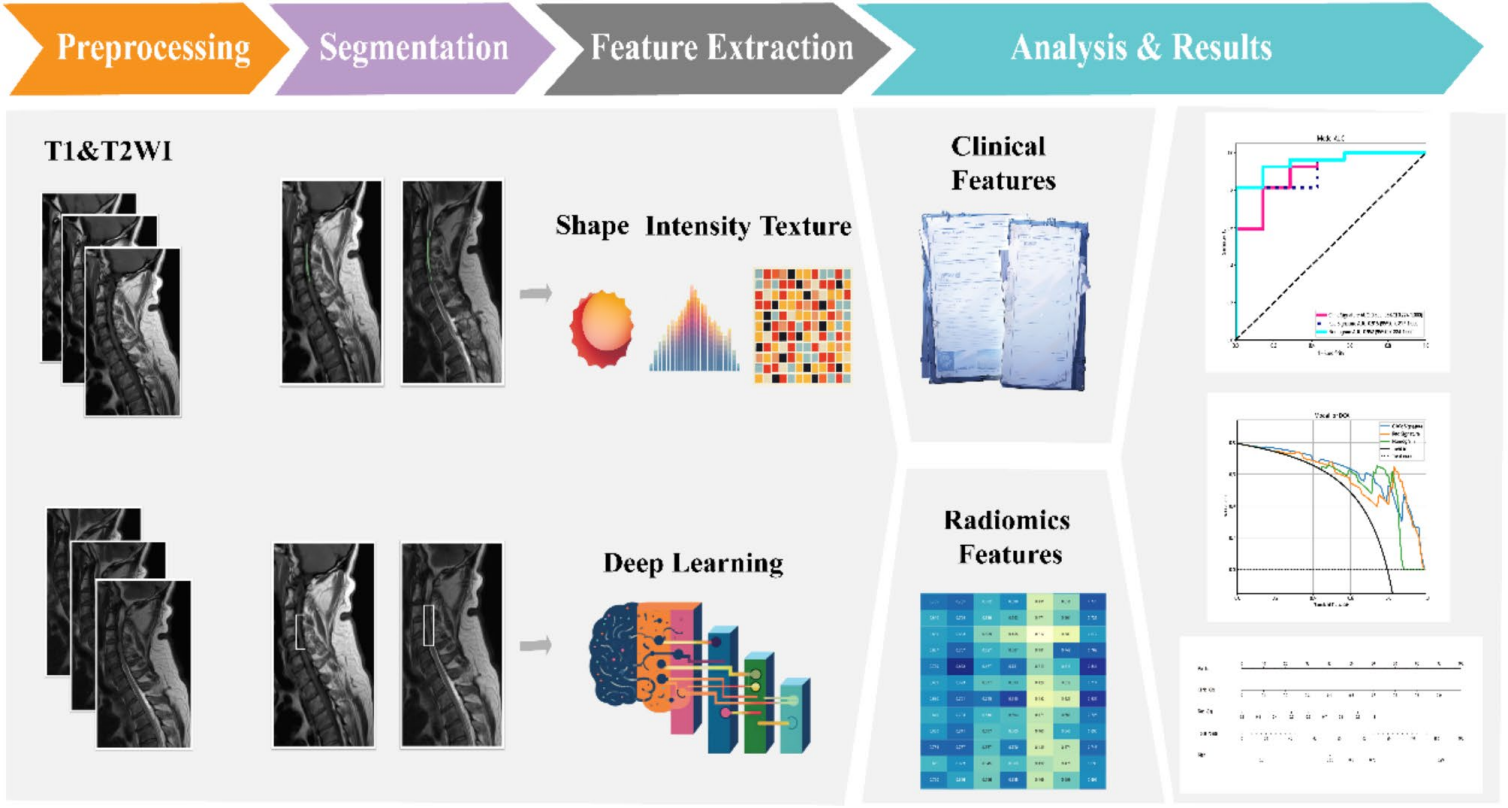
The workflow of model building.

**Fig. 3:**
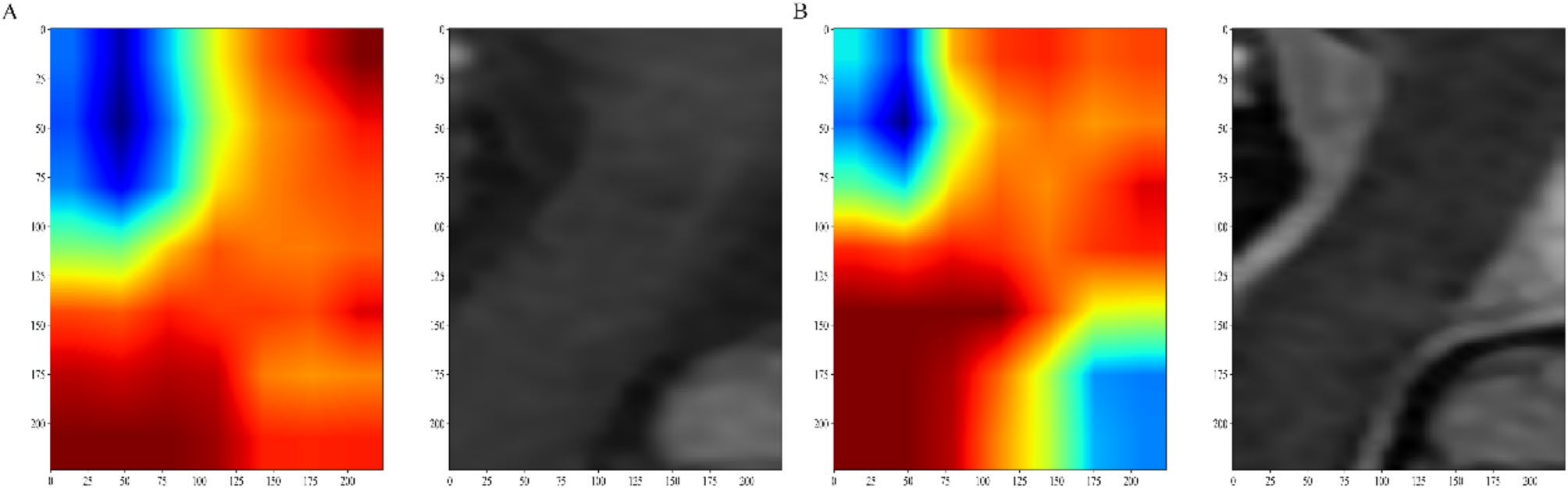
Gradient-weighted Class Activation Mapping (Grad-CAM) visualizations of a patient example. (**A**) T1WI, (** B**) T2WI. The red area shows where the model pays most attention.

**Fig. 4:**
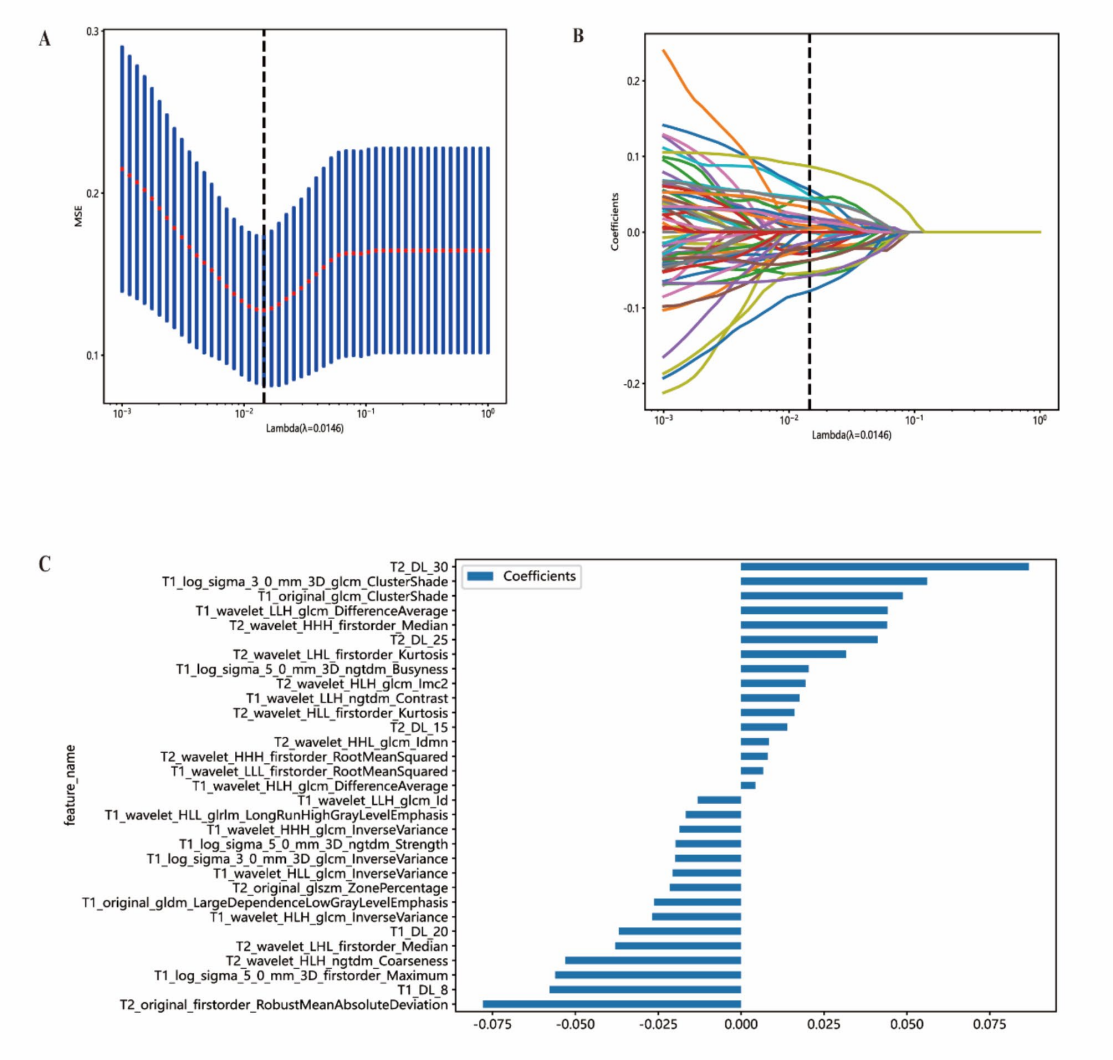
Figures of LASSO regression. (**A**) Mean square error of 10-fold validation. (**B**) Lasso path plot of the best-performance model in the training set. (**C**) Spearman correlation coefficients between features were calculated, and 31 features with correlations were retained.

**Fig. 5:**
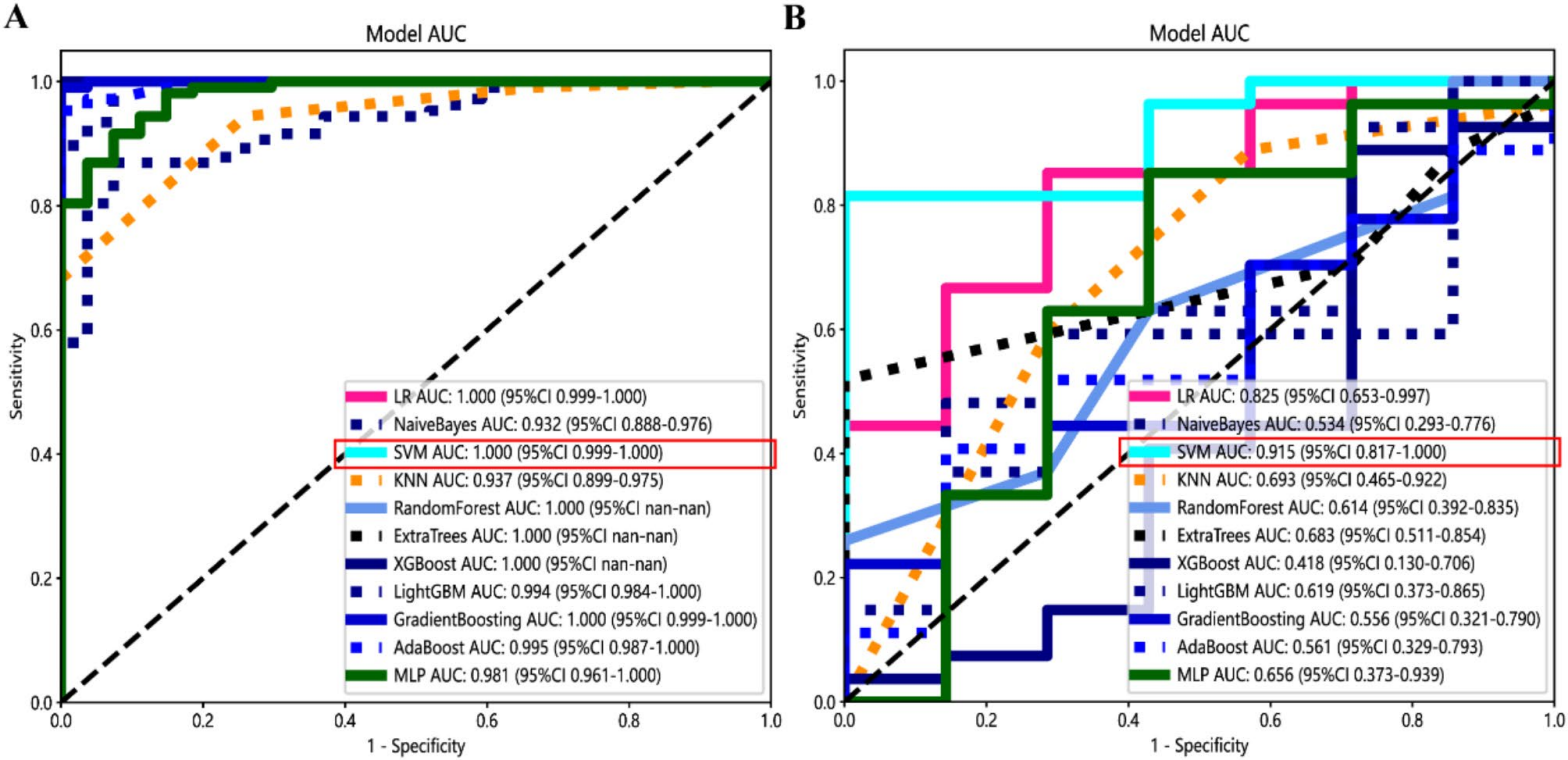
Receiver operating characteristic curves of different classifiers in the training set (**A**) and testing set (**B**). The SVM model got the highest AUC value.

The study workflow is shown in Fig. 2. We extracted features by pyradiomics and ResNet152. Figure 3 shows a class activation mapping visualization example. Following feature extraction, the LASSO algorithm (Fig. 4A and B) was used to filter out handcrafted and deep learning features with non-zero coefficients, ultimately reducing the dimensionality for final 31 features (Fig. 4C). These final features were derived from a combination of 25 handcrafted radiomic features and 6 deep learning-based features(shown in Supplementary Table 3). Each feature’s source sequence and extraction method are detailed in the table. These final features were input into various machine learning classifiers. The SVM classifier achieved the highest AUC, with 1.000 in the training set and 0.915 in the testing set (Fig. 5; Table 3). So we choose the SVM as the classifier of the radiomics model.

Analysis in the retrospective cohort revealed that age, diabetes, and treatment were independent clinical risk factors (Table 2). Comparisons of smoking history, drinking history, hypertension, cardiovascular disease, traumatic brain injury, and injury site revealed no significant differences between different prognosis groups (*p* > 0.05). And significant differences in age, diabetes, and admission ASIA were noted between the groups (*p* < 0.05). A clinical signature, comprising age, hypertension, and treatment, was constructed to develop a clinical model (Table 3). LR was chosen for the clinical model due to its simplicity, interpretability, and suitability for analyzing linear relationships commonly observed in clinical features.

**Table 3:** Main consequences of models.

Model	Dataset	Accuracy	AUC	Sensitivity	Specificity
Clinical	Training	0.882	0.866	0.981	1
	Testing	0.858	0.899	0.778	1
	Validation	0.676	0.754	0.667	0.714
Radiomics	Training	0.985	1.000	0.963	0.444
	Testing	0.824	0.915	0.963	0.571
	Validation	0.794	0.720	0.889	0.429
Combined	Training	0.853	1.000	1	0.839
	Testing	0.978	0.952	1	0.889
	Validation	0.824	0.815	0.852	0.714

**Fig. 6:**
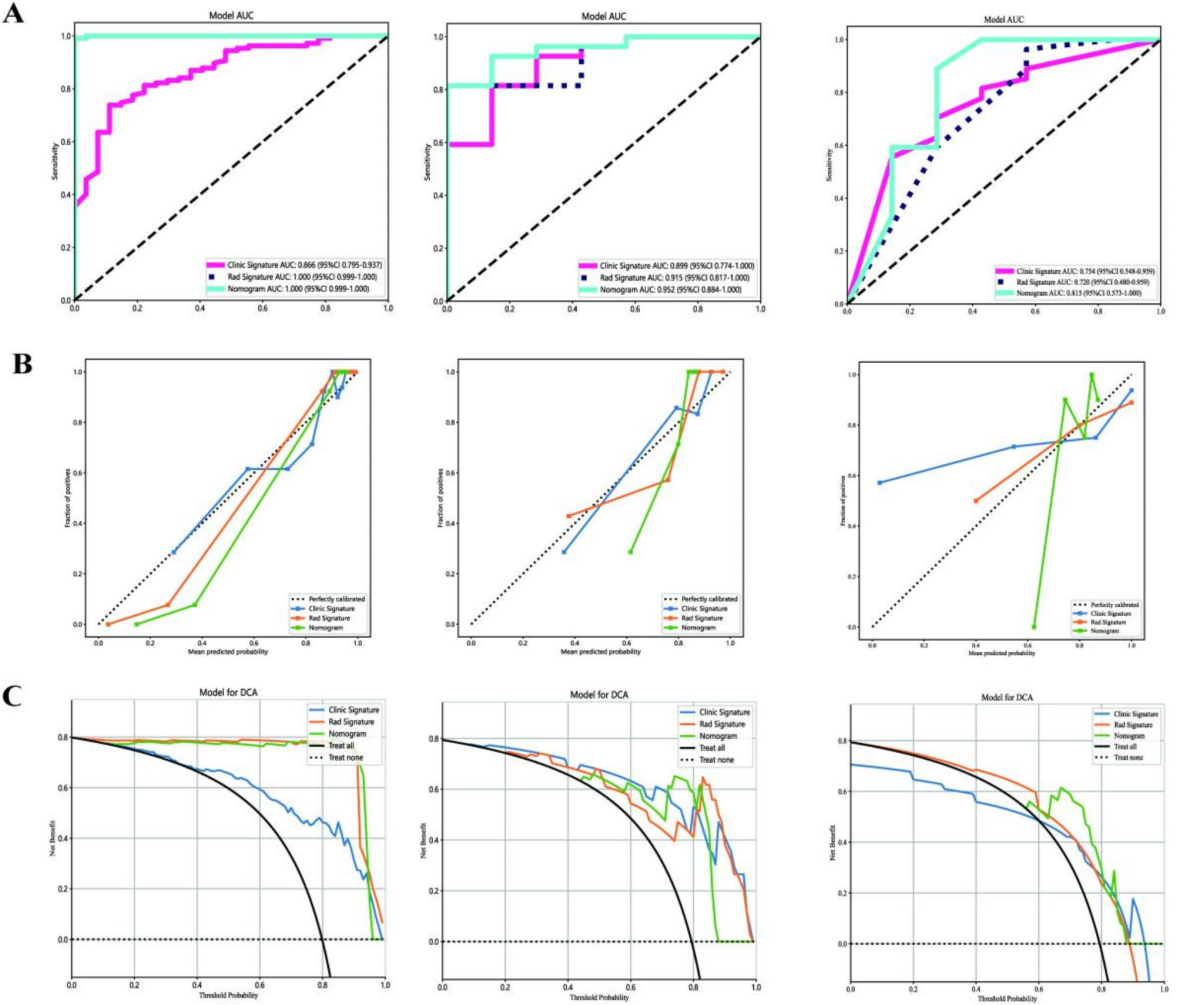
Results of the three models: (**A**) Receiver operator characteristic curves of the 3 models for prediction in the training and testing datasets. (**B**) The calibration curve of the 3 models. (**C**) The decision curve analysis of the three models of the training and testing datasets. Left: Training dataset; Mid: Testing dataset; Right: Testing dataset.

**Fig. 7:**
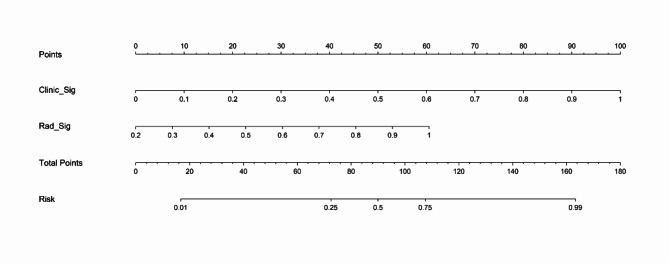
The nomogram combing clinical and radiomics signatures for predicting the prognosis of the cSCI.

A combined model integrating radiomics and clinical features demonstrated excellent performance, with an AUC of 1.000 in the training set, 0.952 in the testing set and 0.815 in the validation set(Fig. 6A; Table 3). Diagnostic accuracy, sensitivity, and specificity for the three models are presented in Table 3. Calibration curves indicated that the combined model’s predicted prognosis closely matched actual outcomes in both datasets (Fig. 6B). Decision curve analysis (DCA) further highlighted the improvement in the combined model across both datasets (Fig. 6C), showing superior performance when the threshold probability ranged from 1 to 99%. We developed a nomogram to visualize the combined model (Fig. 7), allowing for the calculation of risk by summing the points for each variable along the corresponding axis.

## Discussion

This study presents a combined model of radiomics and clinical models that can effectively assess the daily living functions and quality of life of cSCI patients six months post-injury. It also highlights the potential of radiomics and other artificial intelligence technologies in developing personalized treatment plans for cSCI patients.

In this study, selecting an appropriate quantitative metric is crucial for accurately predicting the prognosis of cSCI patients. Quantitative metrics provide objective, reproducible evaluation standards that simplify complex clinical information, facilitate statistical analysis and model construction, and enhance the generalizability and verifiability of research results. We chose the International Association of Neurorestoratology (IANR) score as the metric because it covers multiple dimensions, including motor function, sensation, and activities of daily living, comprehensively reflecting the patients’ rehabilitation status^7^. The sensitivity and specificity of the IANR score ensure accurate capture of functional changes, improving the precision of prognosis prediction. Its widespread use in numerous studies has demonstrated its reliability and validity, making our research results more credible^22,23^. Additionally, the intuitive numerical value of the IANR score helps doctors and patients better understand prognostic information, promoting rehabilitation and treatment work. Therefore, the use of the IANR score not only enhances the scientific rigor and accuracy of the model but also provides reliable guidance for clinical practice.

In terms of imaging feature extraction, we employed two methods. First, we delineated VOI of the injury lesion and used traditional radiomics techniques for feature extraction, as intramedullary pathology is closely related to patient recovery. Accurate extraction of these features is vital for prognosis prediction. Second, we applied deep learning techniques to extract features from the injury site and adjacent anterior and posterior regions of the spinal canal. In the deep learning process, we used the ResNet network and transfer learning techniques. The ResNet network, with its depth and efficient feature extraction capabilities, enabled us to identify and analyze imaging features more accurately. The introduction of transfer learning further improved the model’s performance, making it better suited to the specific conditions of different patients. Additionally, we fully considered the patients’ basic clinical conditions and integrated imaging features with clinical data to develop a comprehensive predictive model. This model relies not only on imaging features but also incorporates clinical information, thereby improving the accuracy and reliability of prognosis prediction. Ultimately, by combining clinical and imaging data, we constructed a holistic predictive model that provides robust support for clinical practice.

Previous studies have been limited by using only clinical data and empirical imaging features^24,25^ or some non-routine inspection^26,27^, restricting their comprehensiveness. Moreover, some studies focused more on local functions, such as walking ability^28^ or the recovery of specific muscles^29^. Based on clinical needs, our study aims to provide clinicians with an overall prognosis expectation for patients before treatment. Our research used MRI T1-weighted and T2-weighted sequences, which have good generalizability and significant applicability in future studies. Our model was effective across different MRI scanners (vendors and field strengths), laying a solid foundation for future multicenter research.

However, this study has limitations. Traditional MRI struggles to reveal molecular information beneath the macroscopic level, such as axonal and myelin preservation, which may be closely related to post-injury recovery. Thus, it is challenging to provide an interpretable biological mechanism for the model. Additionally, other MRI techniques or sequences, such as diffusion-weighted imaging (DWI), which can achieve tractography^30^, could better judge the condition of spinal cord injury. However, challenges such as volume effects in spinal cord imaging, metal artifacts, and the impracticality of requiring patients to remain precisely still for long periods at high resolution necessitate further technological advancements for widespread application. We also acknowledge the need for more external independent validation sets to ensure the generalizability of our findings, which is the next goal of our team.

In summary, this study demonstrates the potential of a combined imaging and clinical model in predicting the prognosis of cSCI patients. This model can provide stratified prognostic assessments for cSCI patients, assist clinicians in patient consultation and guiding treatment and rehabilitation decisions, and potentially improve the design of future treatment plans.

## Electronic supplementary material

Below is the link to the electronic supplementary material.

Supplementary Material 1

## Data Availability

The source code supporting the conclusions of this article is available in the Zenodo repository, accessible via [DOI: 10.5281/zenodo.11499435] (https://doi.org/10.5281/zenodo.11499435). The repository ensures open access to the code under the Creative Commons Attribution License, which permits unrestricted use, distribution, and reproduction in any medium, provided the original work is properly cited. Data supporting the findings of this study are not publicly available due to restrictions related to Zhongda Hospital Neurosurgery Department data governance. Further inquiries can be directed to the corresponding author.

## Abbreviations

AUC: Area Under the Curve
cSCI: Cervical spinal cord injury
DCA: Decision curve analysis
ExtraTrees: Extra Trees
GradientBoosting: Gradient Boosting
ICC: Intraclass Correlation Coefficient
IANR: International Association of Neurorestoratology
KNN: K Nearest Neighbors
LASSO: Least Absolute Shrinkage and Selection Operator
LightGBM: Light Gradient Boosting Machine
LR: Logistic Regression
MLP: Multi-Layer Perceptron
MRI: Magnetic Resonance Imaging
NaiveBayes: Naive Bayes
RF: Random Forest
ROI: Region of interest
ROC: Receiver Operating Characteristic
SVM: Support Vector Machine
VOI: Volume of Interest
XGBoost: eXtreme Gradient Boosting
