# Supplementary material for: Establishment and validation of a ResNet-based radiomics model for predicting prognosis in cervical spinal cord injury patients
Source: Sci Rep. 2025 Mar 17;15:9163. doi: 10.1038/s41598-025-94358-7 (PMC11914052; doi:10.1038/s41598-025-94358-7)
Supplement: Supplementary file 1 — Supplementary Material 1 [file 41598_2025_94358_MOESM1_ESM.docx]

**Supplementary Table 1** MRI Parameters of Selected Sequences

| Parameters | Philips Ingenia 3.0T | | Siemens Verio 3.0 T | |
| --- | --- | --- | --- | --- |
|  | T1WI | T2WI | T1WI | T2WI |
| Repetition time(ms) | 470 | 2500 | 1600 | 2480 |
| Echo time(ms) | 474 | 85 | 9.4 | 103 |
| FOV(mm^2^) | 220×255 | 220×258 | 260×260 | 260×260 |
| Matrix | 244×253 | 244×230 | 192×320 | 257×384 |
| Slice thickness(mm) | 3 | 3 | 4 | 4 |
| Slice gap(mm) | 0.5 | 0.5 | 0.4 | 0.4 |

**Supplementary Table 2** Definitions of Baseline Characteristics

| Characteristic | Definition |
| --- | --- |
| Male/Female (n) | The number of participants categorized by gender. "Male" represents the number of male patients, and "Female" represents the number of female patients. |
| Age (±s, years) | The age of participants, presented as the mean (± standard deviation), measured in years. Age is recorded at the time of diagnosis. |
| Smoking history (%) | The proportion of patients with a history of smoking. This includes current smokers and former smokers (whether or not they have quit). Represented as the percentage of total participants. |
| Drinking history (%) | The proportion of patients with a history of regular alcohol consumption. This includes current drinkers and former drinkers (whether or not they have quit). Represented as the percentage of total participants. |
| Hypertension (%) | The proportion of patients diagnosed with hypertension. Hypertension is defined according to standard criteria, systolic blood pressure ≥140 mmHg, diastolic blood pressure ≥90 mmHg, or current use of antihypertensive medication. |
| Diabetes (%) | The proportion of patients diagnosed with diabetes. Diabetes is defined based on standard guidelines, fasting blood glucose ≥7.0 mmol/L or current use of anti-diabetic medication. |
| Cardiovascular disease (%) | The proportion of patients diagnosed with cardiovascular disease. This includes coronary artery disease, heart failure and myocardial infarction. |
| Traumatic brain injury (%) | The proportion of patients with a history of traumatic brain injury (TBI). TBI refers to confirmed cases of brain injury caused by external trauma to the head. |
| Injury site (%) | The anatomical location of the cervical spinal cord injury, classified into the following categories: |
|  | - C1-4: Injury located between cervical vertebrae 1 and 4. |
|  | - C5-T1: Injury located between cervical vertebrae 5 and thoracic vertebra 1. |
|  | - C1-T1: Injury spans a broad region from cervical vertebrae 1 to thoracic vertebra 1. |
| Treatment (%) | The type of treatment received by patients, categorized as follows: |
|  | - Anterior approach: The proportion of patients treated via anterior surgical approach. |
|  | - Posterior approach surgery: The proportion of patients treated via posterior surgical approach. |
|  | - Anterior & Posterior approach: The proportion of patients treated via a combined anterior and posterior surgical approach. |
|  | - Conservative treatment: The proportion of patients managed with non-surgical conservative treatments, such as medication or physical therapy. |

**Supplementary Table 3** Final Set of Features Used in the Model, Including MRI Sequence and Extraction Method

| Feature Name | MRI Sequence | Extraction Method | Description |
| --- | --- | --- | --- |
| T2_DL_30 | T2 | Deep Learning | Feature extracted using ResNet-based deep learning. |
| T1_log_sigma_3_0_mm_3D_glcm_ClusterShade | T1 | Handcrafted | GLCM feature describing intensity variability. |
| T1_original_glcm_ClusterShade | T1 | Handcrafted | GLCM feature describing intensity clusters. |
| T1_wavelet_LLH_glcm_DifferenceAverage | T1 | Handcrafted | Wavelet-decomposed GLCM feature describing average intensity differences. |
| T2_wavelet_HHH_firstorder_Median | T2 | Handcrafted | First-order feature capturing median intensity in wavelet-decomposed image. |
| T2_DL_25 | T2 | Deep Learning | Feature extracted using ResNet-based deep learning. |
| T2_wavelet_LHL_firstorder_Kurtosis | T2 | Handcrafted | First-order feature representing the sharpness of intensity distribution. |
| T1_log_sigma_5_0_mm_3D_ngtdm_Busyness | T1 | Handcrafted | NGTDM feature capturing the intensity change between neighboring pixels. |
| T2_wavelet_HLH_glcm_Imc2 | T2 | Handcrafted | Wavelet-decomposed GLCM feature describing correlation between intensity pairs. |
| T1_wavelet_LLH_ngtdm_Contrast | T1 | Handcrafted | NGTDM feature quantifying contrast in wavelet-decomposed image. |
| T2_wavelet_HLL_firstorder_Kurtosis | T2 | Handcrafted | First-order kurtosis describing distribution shape in wavelet image. |
| T2_DL_15 | T2 | Deep Learning | Feature extracted using ResNet-based deep learning. |
| T2_wavelet_HHL_glcm_ldmn | T2 | Handcrafted | GLCM feature describing distribution uniformity in the image. |
| T2_wavelet_HHH_firstorder_RootMeanSquared | T2 | Handcrafted | First-order feature describing the root mean squared intensity. |
| T1_wavelet_LLL_firstorder_RootMeanSquared | T1 | Handcrafted | First-order feature capturing intensity variations in wavelet-transformed image. |
| T1_wavelet_HLH_glcm_DifferenceAverage | T1 | Handcrafted | GLCM feature describing the average intensity differences in wavelet-transformed image. |
| T1_wavelet_LLH_glcm_ld | T1 | Handcrafted | GLCM feature quantifying intensity dependency. |
| T1_wavelet_HLL_glrlm_LongRunHighGrayLevelEmphasis | T1 | Handcrafted | GLRLM feature capturing long runs of high-intensity pixels. |
| T1_wavelet_HHH_glcm_InverseVariance | T1 | Handcrafted | GLCM feature quantifying the inverse of intensity variance. |
| T1_log_sigma_5_0_mm_3D_ngtdm_Strength | T1 | Handcrafted | NGTDM feature capturing pixel intensity strength in local regions. |
| T1_log_sigma_3_0_mm_3D_glcm_InverseVariance | T1 | Handcrafted | GLCM feature quantifying local texture homogeneity. |
| T1_wavelet_HLL_glcm_InverseVariance | T1 | Handcrafted | GLCM feature capturing uniformity of intensity distribution. |
| T2_original_glszm_ZonePercentage | T2 | Handcrafted | GLSZM feature describing the percentage of homogeneous intensity zones. |
| T1_original_gldm_LargeDependenceLowGrayLevelEmphasis | T1 | Handcrafted | GLDM feature capturing large dependence on low-intensity gray levels. |
| T1_wavelet_HLH_glcm_InverseVariance | T1 | Handcrafted | GLCM feature capturing the inverse of intensity variance in wavelet-decomposed image. |
| T1_DL_20 | T1 | Deep Learning | Feature extracted using ResNet-based deep learning. |
| T2_wavelet_LHL_firstorder_Median | T2 | Handcrafted | First-order feature capturing median intensity in wavelet-decomposed image. |
| T2_wavelet_HLH_ngtdm_Coarseness | T2 | Handcrafted | NGTDM feature describing the coarseness of image texture. |
| T1_log_sigma_5_0_mm_3D_firstorder_Maximum | T1 | Handcrafted | First-order feature capturing the maximum intensity in the region. |
| T1_DL_8 | T1 | Deep Learning | Feature extracted using ResNet-based deep learning. |
| T2_original_firstorder_RobustMeanAbsoluteDeviation | T2 | Handcrafted | First-order feature quantifying robust deviation from the mean intensity. |
